# Patient-driven research priorities for patient-centered measurement

**DOI:** 10.1186/s12913-024-11182-x

**Published:** 2024-06-14

**Authors:** A. Fuchsia Howard, Linda Warner, Lena Cuthbertson, Richard Sawatzky

**Affiliations:** 1 School of Nursing, The University of British Columbia, T201-2211 Wesbrook Mall, Vancouver, V6T 2B5 BC Canada; 2 Office of Patient Centred Measurement, British Columbia, Ministry of Health, 1190 Hornby Street, 341F, Vancouver, BC V6Z 2K5 Canada; 3 School of Nursing, Trinity Western University, 22500 University Drive, Langley, BC V2Y 1Y1 Canada; 4 Centre for Health Evaluation & Outcome Sciences, St. Paul’s Hospital, 588-1081 Burrard Street, Vancouver, V6Z 1Y6 Canada; 5 Sahlgrenska Academy, University of Gothenburg, Medicinaregatan 3, Box 400, Gothenburg, 405 30 Sweden

**Keywords:** Patient-reported outcome measures, Patient-reported experience measures, Patient-centered measurement, Measurement, Research priorities, Patient engagement, Qualitative

## Abstract

**Background:**

Patient-centred measurement (PCM) emphasizes a holistic approach wherein the voices of patients are reflected in the standardized use of patient-reported outcome and experience measures and are represented throughout the continuum of measurement activities. Given the challenges of routinely integrating patient self-reports into clinical care decisions, the perspectives of all healthcare system stakeholders, especially patients, is necessary to advance the science of PCM. The purpose of the analysis we report on here was to identify patient-driven research priorities for advancing the science of PCM.

**Methods:**

We analyzed data from seven focus groups that were conducted across British Columbia, Canada and that included a total of 73 patients, using qualitative inductive analysis and constant comparative methods.

**Results:**

We found that the patients conveyed a desire for PCM to contribute to healthcare decisions, specifically that their individual healthcare needs and related priorities *as they see them* are always front and centre, guiding all healthcare interactions. The patients’ commentaries highlighted intersecting priorities for research on advancing the science of PCM that would help transform care by (1) enhancing the patient-provider relationship, (2) giving voice to patients’ stories, (3) addressing inclusivity, (4) ensuring psychological safety, (5) improving healthcare services and systems to better meet patient needs, and (6) bolstering healthcare system accountability.

**Conclusions:**

These priorities provide direction for future research efforts that would be positioned to make progress towards better health, better care, and better use of resources for individuals and for society.

## Background

Patient-reported outcome and experience measures (PROMs and PREMs) are tools used to capture the impact of illness on a person’s health and quality of life (PROMs) and their experiences of care (PREMs) [1]. PROMs are self-report instruments used to obtain appraisals of individuals’ health outcomes relevant to their quality of life (e.g., physical and mental health, including symptoms, functional status, and other aspects of psychological, social, and spiritual well-being) [2]. PROMs have been used extensively in research for numerous health conditions, with evidence suggesting PROMs promote improvements in patient-clinician communication and clinical decision-making [3–9]. PROMs also show promise when findings are applied to efforts to improve healthcare utilization [10], quality of life [9], clinical outcomes, and even survival [11–13]. Though PROMs have largely been used in research, they are increasingly collected in clinical settings at the point of care [8]. PREMs are self-report surveys or questionnaires that measure a patient’s perceptions of various elements of the healthcare and services they received [14]. PREMs seek to record what occurred to a patient, from their perspective, and are typically structured around specific dimensions recognized as important to patients [14, 15].

Despite the potential benefits of PROMs and PREMs, much remains to be learned about the experiences and outcomes that matter most to patients to drive the development and use of these measures in different disease, clinical, and geographic contexts. Moreover, challenges to routinely integrating PROMS and PREMS into clinical care and healthcare service delivery have been identified, including logistical concerns, measurement challenges, technological barriers, and a lack of focus on the use of data [16–18]. Efforts to overcome these challenges have included the development of user guides and recommendations by the International Society of Quality of Life and the use and tailoring of conceptual models and frameworks specific to implementation (i.e., the Consolidated Framework for Implementation Research, Integrated Framework for Promoting Action on Research Implementation in Health Services) [19–21].

The term patient-centred measurement (PCM) has emerged to emphasize a holistic approach wherein the voice of patients is not only reflected in standardized measurement tools (e.g., PROMs and PREMs), but also is represented throughout the continuum of measurement activities with patients meaningfully involved during all stages (i.e., design and selection of instruments, reporting, interpretation, and use of the data) [22]. The American Institutes for Research [23] conceptualized PCM as healthcare measurement that is driven by patients’ expressed preferences, needs, and values that informs progress toward better health, better care, and lower costs, wherein “*health care measurement*” referred to any type of health, health status, or healthcare-related measurement and the word, “measurement”, indicated a focus beyond developing measures. PCM additionally requires that patients be engaged at all stages of designing and implementing activities for measuring aspects of what matters about their health and healthcare experiences. Reflecting on prior PCM definitions, the PCM Methods Cluster of the British Columbia (BC) Support for People & Patient-Oriented Research & Trials Unit (BC SUPPORT Unit – part of the Canadian Institutes of Health Research Strategy for Patient-Oriented Research), integrated the focus on experiences and outcomes relevant to patients’ quality of life to contextualize PCM as:

“driven by a desire to understand and improve health-related experiences and outcomes of relevance to patients’ quality of life by identifying, quantifying, and validating what matters to them in order to inform progress towards better health, better care, and better use of resources for individuals and for society” [22].

The PCM Methods Cluster sought to advance the science of PCM, that is, to consider how to transform the data from PROMs and PREMs into relevant information for healthcare providers, health service decision makers, patients, and researchers, to foster action that would result in improved care, patient outcomes, resource utilization, and ultimately better quality of life [24, 25]. To guide their work, this Methods Cluster conducted stakeholder consultation that sought the perspectives of patients, clinicians, researchers, decision makers, and policymakers about methodological concerns that need to be addressed for advancing the science of PCM – that is, advancing the way PCM is done. The purpose of our analysis was to identify patient-driven research priorities for advancing the science of PCM, drawing specifically on the qualitative data arising from the patient focus groups. The term patient in this paper is inclusive of individuals with personal experience of a health issue and informal caregivers, including family and friends [26].

## Methods

### Setting and study recruitment

This research was conducted in the province of BC, Canada, where a publicly funded healthcare system provides universal coverage for medically necessary healthcare services based on patient needs. This study was approved by The University of British Columbia, Research Ethics Board and all participants provided written informed consent. Participants were recruited via employees with accountability for patient experience measurement working in the five regional health authorities, the Provincial Health Services Authority, and the First Nations Health Authority in the province, and included people who had lived experience of the BC healthcare system as a patient or family member and who spoke English. Participants were recruited via the distribution of fliers and posters in healthcare facilities, online forums, and websites.

### Data collection procedures

Two BC SUPPORT Unit investigators (R.S. and L.C.) conducted in-person focus groups with participants during the fall of 2018. Since familiarity with PCM was not an inclusion criterion, each focus group began with an introduction about what PCM entails, including the process of designing surveys, administering the surveys, analyzing the data, reporting results, sharing results and action planning. To provide structure, the focus group guide was constructed to correspond with this introduction, where participants were asked to comment on how they saw patients being involved at each stage of the process and what the participants identified as important to be addressed. The experienced focus group facilitators expressly worked together to ensure that participants had the opportunity to share their perspectives about patient priorities at each stage of the process. They also actively sought diverse perspectives by fostering a safe environment for expressing opinions and encouraging individual input from all participants. Apart from focusing on each stage of the process, all questions were intentionally open-ended, not leading, and loosely followed a semi-structured guide (see Table 1). The focus groups lasted between 60 and 120 min, were digitally recorded, and transcribed verbatim. A total of 10.5 h of transcribed focus group data were collected.

**Table 1:** Focus group guide

The first step, “Designing Surveys or Assessment Tools,” is where we plan the design of our survey or assessment tool. For example, how will the assessment be carried out? How will we recruit participants? What questions will we ask participants? Will we mail a survey, conduct focus groups, or will we conduct face to face interviews? These are all questions that are considered at the design stage. 1. How do you see patients being involved at this stage? a. Optional probe: What about patients being involved in creating the questions that we ask patients? 2. If you could tell the people who are conducting this process one thing that they should focus on, what would that be?
The second step “Collecting Information or Data,” is where we go out and collect information from participants. This might involve calling participants and asking them questions over the phone, asking participants to complete an online or paper survey, or conducting an in-person interview or focus group. 3. How do you see patients being involved at this stage? a. Optional probe: How do we ensure that the people that we talk to are as representative of the diverse population of BC as possible? b. Optional probe: How about translating the question for people who don’t speak English or come from a different ethnic background? c. Optional probe: How about if we have questions for children or youths? d. Optional probe: Are there other specific groups of people who you think we should be asking questions? (e.g., maternity, substance users, seniors? ) 4. If you could tell the people who are conducting this process one thing that they should focus on, what would that be?
This third step, “Processing and Analyzing Information or Data”, is where we have finished collecting information and we want to organize that the raw data (numbers or comments) into information. For example, if 14,076 individual patients who visited an emergency department answered a question with 4 response options…how will we group or cluster the answers? 5. How do you see patients being involved at this stage? 6. If you could tell the people who are conducting this process one thing that they should focus on, what would that be?
*The next segment, “Reporting Results of Analyses” is the stage where we have finished analyzing the information and now want to summarize the findings of our analysis to report back to patients and healthcare providers. Here, we decide on what reports we will publish, and how the results will be displayed and formatted.* 7. *How should patients be involved at this stage?* a. *Optional probe: Should patients be involved in interpreting the results of the data (i.e., figuring out what the data tells us? )* 8. *If you could tell the people who are conducting this process one thing that they should focus on, what would that be?*
*The fifth step, “Sharing Results”, is where we communicate the information we have obtained. Here, we decide who we will communicate with, who are the audiences?* 9. *How should patients be involved at this stage?* a. *Optional probe: In your opinion, who should be the audience or audiences for these reports?* b. *Optional probe: In your opinion, what information should be shared with doctors and other healthcare providers?* c. *Optional probe: In your opinion, should the information provided by a patient about their experiences with the healthcare system be kept with the patient’s medical records?* d. *Optional probe: In your opinion, what information should be shared with patients?* 10. *If you could tell the people who are conducting this process one thing that they should focus on, what would that be?*
*The last segment, “Action Planning”, represents the stage where we want to make the results as useful as possible to make a difference in the health care system (society) and to patients (individuals).* 11. *How should patients be involved at this stage?* a. *Optional probe: What kind of actions do you think healthcare providers can take to improve patient experiences?* b. *Optional probe: What kind of actions do you think patients can take to improve their future healthcare experiences?* 12. *If you could tell the people who are conducting this process one thing that they should focus on, what would that be?*
13. *Which of the points from our discussion matter most to you.* 14. *Can anyone think of anything that is missing from these steps or our discussion?* 15. *Are there any steps here that you see as being more important than other steps?* 16. *Are there any steps in which you believe that patients could not have a role in?*

### Data analysis procedures

We analyzed the focus group data using inductive, thematic analysis, and constant comparative methods [27, 28]. Two investigators not involved in conducting the focus groups (A.F.H. and L.W.) highlighted what appeared to be of greatest importance to the patients within and across the focus group transcripts. Based on readings of the transcripts, we inductively developed an initial coding frame of our interpretations related to patient-driven priorities for research on advancing the science of PCM. The coding frame was revised based on study team deliberations and then applied to transcripts using the qualitative data management software program NVivo™ version 12. We then compared and contrasted [29] analytic codes into broader categories and emergent themes until we were confident that we had captured the predominant ideas, interpretations, and perspectives evident in the focus groups. As a research team we refined these themes and several versions of the written text for clarity and completeness. We identified research priorities that were broadly reflective of the perspectives expressed in the focus groups rather than make comparisons between individuals or groups in the focus groups.

## Results

A total of 73 patients from across seven focus groups participated. The average age was 59 years (range 21–88), and the majority identified as female (72.6%), with greater than a high school education (69%), married (54%), and White (70%), though individuals who were Black, Indigenous, and/or Asian also participated (see Table 2).

**Table 2:** Focus group participant demographics

		Total *n* = 73
**Age (yrs)**		
	Mean	59
	Range	21–88
**Sex**		
	Male	20 (27.4%)
	Female	53 (72.6%)
**Ethnicity**		
	Black	2 (2.7%)
	Chinese	5 (6.8%)
	Indigenous	9 (12.3%)
	South Asian	5 (6.8%)
	West Asian	1 (1.4%)
	White	51 (70%)
**Highest Education**		
	Elementary School	1 (1.6%)*
	High School	17 (27.9%)*
	Some college	18 (29.5%)*
	Baccalaureate Degree	12 (19.7%)*
	Master’s Degree	0 (0%)*
	Doctoral Degree	12 (19.7%)*
	Other	0 (0%)*
	No answer	13 (17.8%)
**Marital Status**		
	Single	0 (0%)*
	Never married	11 (18%)*
	Common-law	1 (1.6%)*
	Married	33 (54.1%)*
	Divorced	8 (13.1%)*
	Widowed	5 (8.2%)*
	Separated	2 (12.7%)*
	No answer	13 (17.8%)
**Health Authority**		
	Fraser Health Authority	10 (13.7%)
	First Nations Health Authority	6 (8.2%)
	Vancouver Coastal Health Authority	11 (15.1%)
	Vancouver Island Health Authority	12 (16.4%)
	Interior Health Authority	13 (17.8%)
	Northern Health Authority	9 (12.3%)
	Provincial Health Services Authority	12 (16.4%)

*Total calculation of percentage does not include PHSA population as this information was not collected (*n* = 61)

**Fig. 1:**
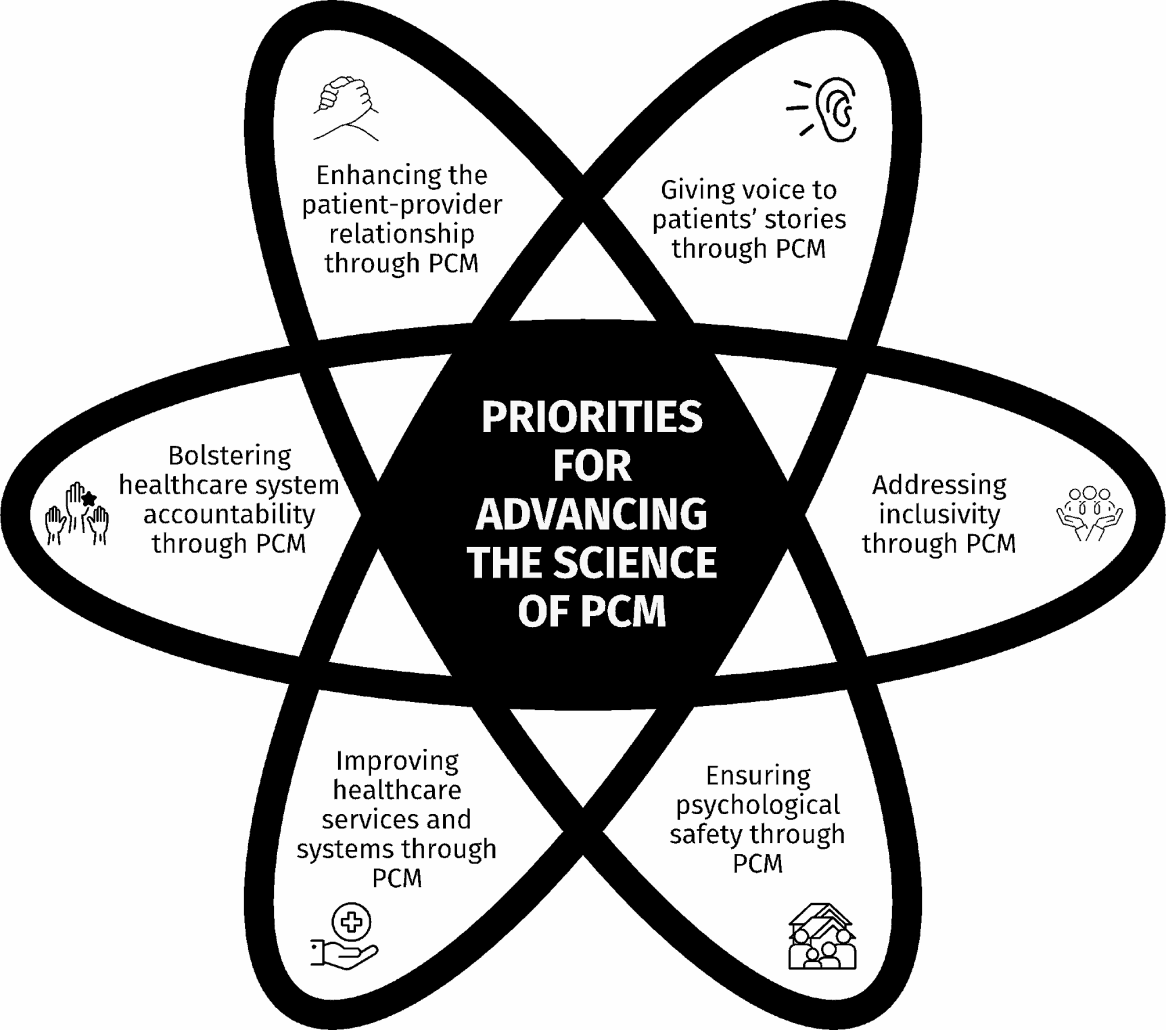
Intersecting priorities for research for advancing the science of PCM

Overall, the patients conveyed a desire for PCM (their self-reports of their experiences and their outcomes) to contribute to healthcare decisions, specifically that their individual healthcare needs and related priorities *as they see them* are always front and centre, guiding all healthcare interactions. The patients’ commentaries highlighted intersecting priorities for research on advancing the science of PCM that can be seen to collectively lead to the generation of the evidence necessary to address what matters most to them as individuals. The patients saw these priorities as having the potential to help transform care by: (a) enhancing patient-provider relationships, (b) giving voice to patients’ stories, (c) addressing inclusivity, (d) ensuring psychological safety, (e) improving healthcare services and systems to better meet patient needs, and (f) bolstering healthcare system accountability (see Fig. 1). Across these priorities, the patients articulated the importance of engaging with patient and family partners as research team members right from the research/project idea generation stage and throughout all research phases. Moreover, the patients described the need for capacity building for both patients and researchers in the form of education and training that fosters patient engagement in PCM research.

### Enhancing patient-provider relationships through PCM

A priority for research on advancing the science of PCM apparent across focus groups was the desire for evidence to guide how PCM could be integrated into their care to enhance a patient’s relationship with their provider. The patient-provider relationship was greatly valued, with the desire for PCM data to strengthen trust, respect, and reciprocity. PCM was framed as a potential mechanism by which a provider could be made aware of a patient’s individual priorities, which might differ from the priorities of other people or groups, so that what matters most to the individual patient is addressed in all interactions. Given the personal nature of questions about health and healthcare experiences, the patients emphasized that it takes time to build a relationship to the point where a patient sufficiently trusts their provider to feel safe and give honest responses. The significance of trust in the patient-provider relationship that was communicated by the patients draws attention to the importance of finding ways of establishing, sustaining, and enhancing trust through PCM approaches.

*“If you [healthcare provider] have a scripted survey, you are kind of asking things that you think that are important to them [the patient], not what they would like to share with you. And sometimes, those conversations cannot happen right away. In other work, we talk about how it might take a little bit of time to build a relationship for somebody to have the confidence and the trust in that relationship to be able to tell you or to be able to start the work that needs to happen.” – Focus Group #2*.

Further, the patients wanted PCM to be the impetus for helping their provider(s) genuinely hear their individual concerns and engage in dialogue to build a respectful partnership.

*“We are emotional; we engage in the person. This person’s got our life and health in their hands, and we want them to like us. We want this to sort of be almost a spiritual relationship. If you have cancer or a life-threatening disease, this is very heavy stuff.” – Focus Group #1*.

The patients also advocated for a personal commitment from providers and the healthcare system for sustained and respectful engagement. This respectful engagement appeared to be a key aspect of PCM that bolsters greatly valued patient-provider connections. Further, the patients wanted some assurances that their information was going to be used constructively, including contributing to positive changes to healthcare services and systems.

*“Doing something like measuring the care and wanting to make any kind of changes, from my perspective, the first thing would be commitment, not just from you guys who are going to be trying to make these changes, but from the people you are working with as well, from the doctors and the nurses and from the health practitioners. If they are going to say, “Yes, we want to be a part of this, and we want to effect change. I am committed to this, and I will always be.” – Focus Group #2*.

### Giving voice to patients’ stories through PCM

The patients’ remarks across focus groups suggested that the generation of research evidence that facilitates giving voice to patients’ stories was a priority. Patient perspectives, collected through PCM tools that complement and supplement clinician-reported indicators and data were framed by the patients as key to communicating patients’ experiences and health status. Several of the patients suggested that there are some aspects of human experience that elude numerical measurement but that nonetheless, measures are vital to capture and communicate to their clinicians, to policymakers, and to people who evaluate healthcare services or programs. Patient stories, their narratives, their poems, the qualitative data, free-text comments collected through surveys and patient journey mapping were all suggested as ways to both “humanize” and contextualize, or illustrate, numerical data. These narrative and qualitative data were positioned as a means of enabling patients to communicate their priorities, in part because numerical data could “fall on deaf ears,” while story-like formats were considered by participants to be a more effective means of reaching various audiences. The patients did not want to be seen as “just a data point,” and believed their stories could help foster an appreciation of patients’ experiences and enhancement of connection with providers and peers.

*“If you rattle off a bunch of numbers to me, it does not mean anything, but if that is encompassed in a story, then it is more relatable. It is something I will remember. I will not remember that 92% of people said “Blah, blah, blah”, but I will remember the story. I think that is being a little bit more strategic about it because you are also able to connect with your audience, whether it’s the doctors or the patients. I think being able to connect with the stories will mean more, and you will get more out of it.” - Focus Group #7*.

### Addressing inclusivity through PCM

The patients described PCM that addresses inclusivity as a priority, suggesting this area is a priority for research on advancing the science of PCM. They referred to inclusivity when speaking about individuals and groups who experience marginalization or vulnerability related to their age, gender, sexual orientation, ability, and health status, but also to social and structural influences of racism, colonialism, sexism, stigmatization, and discrimination. They highlighted how such individual differences and the intersections of social and structural factors create different health and healthcare experiences for people, which should be considered in PCM. Suggestions for enhancing inclusivity focused on ensuring a diversity of individuals and perspectives are represented through PCM and including family and communities in PCM.

#### Ensuring a diversity of individuals and perspectives are represented through PCM

The patients rejected a one-size-fits-all approach, highlighting accessibility and representation of diversity as conditions for inclusive PCM. They articulated the importance of ensuring accessible formatting and timing of assessments. There was consensus that both accessible paper and electronic means of responding to measures were necessary to ensure that those who do not use digital communication can respond. Additional yet undeveloped accessible formats were identified as required to accommodate different patient abilities, such as those with physical, neurocognitive, or other impairments. There was a range of opinions about the optimal timing to collect information reflecting the complexity of individuals’ situations. Providing information during or shortly after a healthcare encounter was deemed necessary for some, while for others, having time to think about their responses was key to ensuring their replies reflected their experiences and priorities.

*“I can speak for my son. He has very bad short-term memory, so if he has been involved in a meeting with someone, sometimes he is able to give an impression right away and a response. He will have just forgotten the whole thing two hours later. With other things, the cognitive process has to take place, and it takes a while. Lots of times, I will speak to him on the phone, and he will not say anything, but then half an hour later, he will say, “Oh, this, this and this,” but it actually took some time for him to process all that.” - Focus Group #7*.

Moreover, the patients highlighted how devising ways to involve individuals to support patients to provide their perspectives was key to ensuring accessibility in PCM, especially for those who experience cognitive challenges or unfamiliarity with providing personal views. Numerous instances were recounted where they assisted others in completing PCMs by reading questions out loud, explaining or interpreting questions, and engaging in discussion or dialogue to ensure the responses accurately represented the patient’s experiences.

*“I was collecting data for the [name] survey… I found it challenging because there were too many choices for a lot of the clients. So, when they were not sure, they were like, “Oh, we are here somewhere.” Then I would have to breach protocol and kind of give them the options. It is hard to be true to the methodology sometimes when you are in real life with the subjects… I know that you want to discriminate finely amongst responses, but with some populations – and certainly the population in complex care – I found it very challenging for them to be able to sort out all these choices.” - Focus Group #4*.

Beyond support, the involvement of a proxy individual, often a family member or caregiver, was commonly flagged as vital for obtaining accurate and valid information.

Creating ways to ensure PCM is relevant and represents the diversity of individuals who experience a wide range of health challenges was a priority across focus groups. As one patient described, traditional approaches to measurement were not always developed from the perspectives of those for whom a measure was intended.

*“I think society, in general, has a bias that affects all marginalized groups; that we look at somebody in a wheelchair as the perspective of somebody who can walk. We look at gay and lesbian people from the perspective of people who are straight. We look at folks who have disabilities, be they physical or cognitive, from the perception of somebody who is normal, whatever the heck is.” - Focus Group #5*.

A key question was, how can PCM reflect the perspectives and priorities of those experiencing vulnerability or marginalization from their own point of view and not the dominant gaze. To be inclusive, an important focus was to advance approaches for tailoring PCM to the social and cultural context and health literacy of historically excluded individuals.

#### Including family and communities through PCM

The patients drew attention to ways of conceptualizing PCM that were inclusive of family members and communities. Thus, efforts that extend PCM to target the outcomes and experiences of family members and communities appeared to be a priority for research on advancing the science of PCM. This was positioned as key to assessing how health and illness affect the patient’s natural supports, such as “*family members, friends, and loved ones,”* and highlights a shift to care of the family as within the purview of the healthcare system. The patients also explained that PCM assessment and subsequent care should include a focus on communities, and not just individuals or families.

*“We were talking about birth protocol and death and dying protocols. Who is the patient in that situation? If a youth commits suicide in a community, who is the patient in that situation? … I like to learn to approach things from a both hands [two-eyed seeing] perspective and try to balance this out.” - Focus Group #2*.

Thus, PCM that includes family members, community members, and the broader community was endorsed out of recognition of the need for approaches and interventions at all levels.

### Ensuring psychological safety through PCM

The patients commonly expressed concern that the process of collecting and utilization of PCM information has the potential to cause harm to individuals and communities. Potential harms mentioned included difficult or distressing thoughts or emotions, disrespectful or unfair interactions with clinicians and healthcare organizations, and barriers accessing or utilizing healthcare services. As such, factoring safety into PCM was considered crucial. This pertained to how patient responses are gathered (e.g., the way questions are asked, how often, and by whom), the nature of the information collected, and data utilization. The patients articulated an asymmetrical dynamic such that patients and families are largely powerless and vulnerable to reprisal when asked about their care experiences. At the same time, healthcare providers and programs were described as holding positions of power. It was apparent in the patient narratives that this power imbalance is potentially exacerbated in a healthcare context with poor access to primary and specialized services, long procedure waitlists, and dependence on providers for care. The patients described fear of reprisal and recounted numerous instances where they felt they had been punished for providing feedback that was critical of a healthcare provider.

*“If you give negative feedback, you are worried that this feedback might make the program shut down. So, then you feel a little forced or coerced into giving positive feedback. Also, when you give feedback to a doctor or a program, you worry about being recognized… Even if you are anonymous, but you write comments that may be identifying… It has happened to me when I gave feedback, and I received negative consequences… The doctor said this never happened, that I was a liar… So, it made me learn that if I give feedback, that is something that is going to happen to me. Actually, it seems like that project or their program does not want to deal with me at all.” – Focus Group #3*.

If safety is not ensured throughout all aspects of PCM, the patients indicated that, at best, patients would not provide any information or would not provide honest information. At worst, forthcoming patients felt they could be at risk of being inappropriately confronted, treated poorly, or denied care. Thus, devising means by which PCM is conducted, implemented, and interpreted and used in ways that are safe for patients stood out as a research priority. The patients were acutely aware of the challenges of ensuring confidentiality and anonymity when collecting, analyzing, and using the information to improve care. However, solutions to overcome these challenges were considered critical to ensure that patients can provide feedback in a way that does not make them fearful of retaliation, secondary trauma, or loss of trust in providers. Further, appropriate means of integrating supports, champions, and advocates into PCM data collection were highlighted when considering individuals who experience mental health or substance use stigma, dependence for basic needs and activities of daily living (i.e., in extended care), racism, and discrimination. Though primarily discussed as a safety issue, this was also deemed key to obtaining accurate information through PCM.

*“What is a really important factor in collecting information from patients is peer work… Someone that they know and that they can trust… because with a lot of people, that is how you are going to get the truth.” – Focus Group #7*.

### Improving healthcare services and systems to better meet patient needs through PCM

A priority for research on advancing the science of PCM apparent across focus groups was the desire for evidence that provides guidance for how best to integrate PCM to improve services and systems of care. The patients commonly characterized the state of healthcare in BC and Canada as strained. The patients often reported facing challenges accessing primary and specialist care promptly, discussing complex health issues in the limited time available, transitioning from one provider/service to the next, coordinating care between different providers/services, and obtaining follow-up and results from investigations. Without careful consideration of how to integrate PCM into existing healthcare services and systems, the patients expressed concern that existing strains could be exacerbated for both patients and providers.

*“It is a double-edged sword. The physician is going to get as many people in as they can for servicing, and at the same time, the patient is going to want a lot of time, and if they are asking [questions] that they will not completely understand, the physician has got to be patient enough to expand on that and to get them to understand [the questions].” - Focus Group #1*.

As such, the patients advocated for PCM that is responsive to individual patient needs first and foremost, but also promotes an effective and efficient healthcare system. They commented on aspects of PCM that warranted further investigation, including the optimal timing of patient-reported assessments (i.e., before or after a care encounter), the frequency of patient-reported assessments and how a single assessment fits in a person’s overall care journey (i.e., concerning different treatment/therapy or stages of illness), the location where patient’s complete patient-reported assessments (i.e., hospital versus home), and the method of collecting information (i.e., online/remote versus in person). The patients collectively voiced a desire for patient-reported assessments that are integrated in a manner that works well for the patient and the healthcare provider, is coordinated and communicated to enhance continuity of care, and reduce rather than create bureaucracy.

*“One of the main things is coordination between all these different factions. You go to one, and you are told one thing, or you have a result with one person and you assume that this other person, who is deeply involved, will be advised. “No,” and so you have to start all over again. There is no coordination, speaking of a systemic thing, but how many systems are there going to be, and how is that decided? That is a huge thing.” – Focus Group #7*.

The patients implied that integrating patient-reported assessments in ways that improve healthcare services and the system could prevent patients from having to answer the same questions multiple times, inspire patients to complete assessments knowing they will be shared with members of their healthcare team, and streamline the flow of information so that providers have the information they need, when they need it, to address the patient’s health concerns.

### Bolstering healthcare system accountability through PCM

The patients’ remarks across focus groups drew attention to the potential role that PCM could play in bolstering accountability at the healthcare system level. Yet, the patients raised numerous questions about *how* PCM could bolster this accountability and ensure that patient feedback is heard and acted upon, indicating a priority for research on advancing the science of PCM. There were different perspectives among patients who commented on the accountability of individual healthcare providers versus the team, versus the healthcare system, raising questions about whether patients ought to report on their experiences with individual providers, the healthcare team, or the system. Regardless, the patients articulated preferences for clear, transparent, and upfront communication, specifying how information and patient feedback would be used. Some individuals raised concerns about the responsible and ethical use of patient-reported data, such that information is used in the ways intended and agreed on by those providing it. Across the focus groups, it appeared that accountability included ensuring that PCM data are transparently reported to all relevant stakeholders, yet the patients raised questions about how this could be done.

*“How do we actually display the information in a way that is going to be useful for many different audiences? Everything from the general public, people like you, patients, as well as policymakers, government leaders, clinicians. What… how do we display the information in the way that is most useful for each audience? Then how do we actually disseminate the results? How do we share them and make them available?” - Focus Group #6*.

Aspects of PCM that appeared key to bolstering healthcare system accountability included taking action, making tangible changes based on the information, and demonstrating to patients how PCM data led to action and changes. Several of the patients commented on their frustration having provided input that was ignored or they felt made no difference.

*“There is a bit of a lack of trust in the system, and it is very difficult to engage people when they do not feel that their voice is respected and listened to, or that there will be any results. There is lots and lots of information that gets asked, and not just within healthcare, but in many different places in our lives. Where we are asked to give information, and we never see results. And so, it is very difficult. So that is one thing that kind of needs to change.” - Focus Group #6*.

Thus, the patients wanted to know how their input would meaningfully inform “actionable” changes, ultimately increasing the accountability of the healthcare system to the patient.

### Intersection of themes

Together, the six themes intersected to illustrate the prioritization of research in developing and implementing comprehensive PCM, transforming individual patient care, and fostering a patient-centred healthcare system. Enhancing patient-provider relationships through PCM highlighted trust, respect, and reciprocity, which might be further enhanced with patients’ stories that provide a more holistic view of patient experiences. Addressing inclusivity through PCM emphasized the need for accessible and adaptable approaches to various patients, which could then in turn, foster respect and understanding. Similarly, psychological safety through PCM appeared crucial for trusting patient-provider relationships, allowing patients to provide honest feedback without fear. Additionally, inclusive representation of diverse patient experiences might enhance psychological safety and promote responsive healthcare services and systems. Psychological safety that fosters honest patient feedback might further provide a mechanism to improve healthcare services and systems and bolster healthcare system accountability through the transparent and actionable use of PCM data. Participants emphasized that patient input must lead to tangible changes in healthcare, demonstrating their voices are heard and valued. Moreover, accountability might help ensure PCM data are used responsibly, ethically, and effectively, fostering trust in the patient-provider relationship and encouraging patient engagement throughout PCM. Together, these interconnected themes could potentially help create a responsive, inclusive, and trust-based healthcare environment, and prioritize patient needs and experiences while ensuring continuous improvement and accountability.

## Discussion

To date, PCM research agendas have primarily focused on PROMs and have been driven by researchers and healthcare providers, with some formal priority-setting efforts inclusive of more diverse stakeholders [30–33]. Though some of these efforts included patient perspectives, rarely have patients’ opinions about priorities for research to advance the science of PCM been the focus, as they were in the focus groups across the province of BC Canada, that led to the findings presented here. What stood out in our findings was the ability of the patients to articulate how important the *function* of PCM is in the context of an individual’s health and healthcare. That is, the patients’ commentaries suggested the prioritization of research wherein PCM could potentially transform an individual patient’s care and the broader healthcare system.

This focus on the function of PCM, inclusive of the ways in which PCM could influence an individual patient’s care and the responsiveness of the healthcare system to their needs, contrasts with the proliferation of PROMs and PREMs research devoted to developing specific health or experience outcomes, such as symptom severity, patient satisfaction, and quality of life, and evaluating the effectiveness of PROMs and PREMs use on these outcomes. That is, researchers have focussed on whether using specific PROMs or PREMs impacts specific health or experience outcomes. For example, does the use of a pain PROM contribute to a decrease in pain. Our findings suggest that additional investigations, including realist evaluations, are warranted to discern *how*, *for whom*, and *in which circumstances* PCM can be utilized to ensure that what matters to individual patients is prioritized in all healthcare encounters [34]. For example, *how* does the use of a pain PROM influence pain-related conversations with healthcare providers, exploration of the multidimensional consequences of pain of importance to patients, or the offering of various approaches to pain management valued by patients. This aligns with a research focus on understanding and explaining how PCM works, as articulated by Greenhalgh and colleagues [35], to influence the various components of care.

The patients in our research, however, also went beyond the “how” to emphasize the foundational nature of the patient-provider relationship for quality healthcare that meets their individual needs. They advocated for PCM that would enhance this relationship. This finding would align with research efforts that focus on the influence of PCM on various aspects of clinical care, such as communication and shared decision-making. For example, evidence suggests that PCM might increase symptom awareness, prompt discussion, and create opportunities for patients to elaborate on their problems, thus facilitating patient-provider communication [3, 5, 36]. Further, improved communication, the provision of patient-specific information, and a more holistic understanding of the patient’s condition and experiences via PCM might influence the therapeutic relationship between the patient and the provider [6]. Our findings suggest that these aspects of clinical care are important research foci, but enhancing care through PCM could also be investigated from a patient’s perspective, emphasizing concepts such as cultural safety, trust, respect, reciprocity, feeling heard, and partnership.

Further, our findings highlight the importance of data and information that helps providers listen to and give voice to patients’ stories to enhance appreciation of individual experiences and a sense of connection, as also articulated by Cuthbertson et al. [15]. Recognizing the limitations of PROMs and PREMS, some have called for research that enables the collection and utilization of patient narratives in ways that present meaningful insights into patients’ health and quality of life in combination with metric scores [37]. This is a research area wherein many research questions remain. For example, how might patients’ stories or narratives be collected, integrated with numerical data, presented, or used by clinicians [37]?

The patient-driven priority for research for advancing the science of PCM that attends to the diversity of patient abilities and individual needs and ultimately contributes to health equity parallels commentary by others. In their call for inclusive and equitable patient-reported outcomes, Calvert et al. [38] identified barriers to the completion of PROMs, including limited access to technology and burdensome language, formats, or platforms for people with disabilities, low literacy, or in poor health. They also argued that lack of representative PROM data limits understanding of the impact of disease or treatment on patients’ symptoms and quality of life, and thus the evidence to inform clinical care, make regulatory decisions, and inform health policy. Building on evidence of a misalignment between what patients report as important to them about their disease and treatment, and the data typically collected, others have recommended expanding collected data to include all impacts and concerns important to patients, not only health outcomes [39]. What these outcomes are, particularly for those historically and currently not represented in PCM, remains to be determined. Further, integrating inclusivity and safety into PCM will be a complex challenge, warranting research in measurement conceptualization, development, and psychometric testing, PCM implementation, and utilization of information in clinical contexts.

The pragmatics of managing health and illness can be immense for patients and families. Unsurprisingly, the patients in our study greatly valued healthcare services when the system worked for them. They wanted PCM to be integrated such that services would be more responsive to their individual patient needs, culturally safe, effective, and efficient. Numerous barriers and challenges that hamper PCM implementation into existing healthcare services have been identified. These include logistical and technological concerns related to staff workflow, staff time and resource requirements, integration into existing medical records, and processes that make data actionable for clinical care [16]. While healthcare services and systems that work well for staff might also improve patient experiences, this is not guaranteed. Our findings suggest that focusing on the implications of PCM implementation specifically for patients is warranted. Further, there is growing recognition of the value of principles of participatory research and integrated knowledge translation that engages all stakeholders, especially patients, in designing and implementing systems to capture and make PCM actionable [32]. Engaging patients is also critical to developing mechanisms to communicate how PCM will be used, ensuring patients feel safe to provide input that informs actionable changes at a system level, and ultimately increasing accountability to the patient. There is evidence indicating that public reporting of aggregate PROM data improves the quality of patient care [40]. However, there is limited patient-perspective evidence of the benefit of different reporting strategies.

Study results ought to be interpreted in context and with study limitations in mind. This research was conducted in BC, Canada, and thus, study insights are most relevant to places with a publicly funded healthcare system wherein the integration of PCM into clinical care has not been routinely implemented across the healthcare system. The study sample was large for qualitative analysis and focus groups were held in each of the BC health authorities thereby enhancing variation in patient demographics and experiences. However, we could not attribute individual quotes to specific participants with known ethnicity or gender because the data were de-identified to abide by our research ethics approval. Thus, we cannot ensure that the voices of individuals from equity-deserving groups were represented in the data, though representativeness was not the goal of this qualitative research. This research aimed to identify research priorities that were broadly reflected in the study focus groups, and therefore, other individuals and groups likely have somewhat different priorities or perspectives. It is very likely that our results do not represent the full diversity of patient perspectives nor do they represent all groups of people who may have different opinions about research priorities. This might also include patients who do not speak English, who are uncomfortable voicing their opinion, or who experience significant structural vulnerability.

### Implications for research

The study’s findings emphasize the importance of prioritizing PCM research that aims to transform individual patient care and the broader healthcare system. Additionally, the study underscores the foundational nature of the patient-provider relationship for quality healthcare, and the need for research that bolsters the use of PCM as a tool to enhance communication, shared decision-making, and trust. Novel means of integrating patient narratives with numerical data warrant development as a potential strategy to provide meaningful insights into patients’ health and quality of life, helping clinicians appreciate individual experiences and improve therapeutic relationships. Finally, the study highlights the need for research to explore and develop inclusive PCM that addresses the diversity of patient needs and experiences, particularly those from equity-deserving groups, and to engage diverse patients in designing and implementing PCM systems to make healthcare services more responsive and accountable.

## Conclusion

In conclusion, the shift from PROMs and PREMs to PCM holds tremendous promise as a way to orient healthcare and health systems around what matters most to individual patients. The patient-driven priorities for advancing the way PCM is done that we identified in this study underscore patients’ desires for their healthcare needs and related priorities as they see them to always be front and centre, guiding all healthcare interactions. The priorities described in this study provide direction for future research efforts that are grounded in patients’ perspectives and, thus, positioned to truly make progress towards better health, better care, and better use of resources for individuals and for society.

## Data Availability

The datasets analyzed during the current study are not publicly available because they contain information that could be used to identify individual participants who did not provide written informed consent to make data available. It is uncommon to make qualitative data, such as those used in this study, publicly available.

## Abbreviations

PROMs: Patient-reported outcome measures
PREMs: Patient-reported experience measures
PCM: Patient-centred measurement

## References

[1] Nolte E, Merkur S, Anell A, North J. Achieving person-Centred Health systems: evidence, Strategies and challenges. Cambridge University Press; 2020.

[2] Fayers PM, Machin D. Quality of life: the assessment, analysis and reporting of patient-reported outcomes. Wiley; 2015.

[3] Greenhalgh J, Abhyankar P, McCluskey S, Takeuchi E, Velikova G (2013). How do doctors refer to patient-reported outcome measures (PROMS) in oncology consultations?. Qual Life Res.

[4] Kotronoulas G, Kearney N, Maguire R, Harrow A, Di Domenico D, Croy S (2014). What is the value of the routine use of patient-reported outcome measures toward improvement of patient outcomes, processes of care, and health service outcomes in cancer care? A systematic review of controlled trials. J Clin Oncol.

[5] Yang LY, Manhas DS, Howard AF, Olson RA (2018). Patient-reported outcome use in oncology: a systematic review of the impact on patient-clinician communication. Support Care Cancer.

[6] Boyce MB, Browne JP, Greenhalgh J (2014). The experiences of professionals with using information from patient-reported outcome measures to improve the quality of healthcare: a systematic review of qualitative research. BMJ Qual Saf.

[7] Noonan VK, Lyddiatt A, Ware P, Jaglal SB, Riopelle RJ, Bingham CO (2017). Montreal Accord on patient-reported outcomes (PROs) use series–paper 3: patient-reported outcomes can facilitate shared decision-making and guide self-management. J Clin Epidemiol.

[8] Greenhalgh J, Gooding K, Gibbons E, Dalkin S, Wright J, Valderas J (2018). How do patient reported outcome measures (PROMs) support clinician-patient communication and patient care? A realist synthesis. J Patient-Rep Outcomes.

[9] Gibbons C, Porter I, Gonçalves-Bradley DC, Stoilov S, Ricci-Cabello I, Tsangaris E et al. Routine provision of feedback from patient-reported outcome measurements to healthcare providers and patients in clinical practice. Cochrane Database Syst Rev. 2021(10). 10.1002/14651858.CD011589.pub2 PMC8509115 34637526

[10] Howell D, Li M, Sutradhar R, Gu S, Iqbal J, O’Brien MA et al. Integration of patient-reported outcomes (PROs) for personalized symptom management in real-world oncology practices: a population-based cohort comparison study of impact on healthcare utilization. Support Care Cancer. 2020:1–10. 10.1007/s00520-020-05313-3 32020357

[11] Ediebah DE, Quinten C, Coens C, Ringash J, Dancey J, Zikos E (2018). Quality of life as a prognostic indicator of survival: a pooled analysis of individual patient data from Canadian cancer trials group clinical trials. Cancer.

[12] Berg SK, Thorup CB, Borregaard B, Christensen AV, Thrysoee L, Rasmussen TB (2019). Patient-reported outcomes are independent predictors of one-year mortality and cardiac events across cardiac diagnoses: findings from the national DenHeart survey. Eur J Prev Cardiol.

[13] Raffel J, Wallace A, Gveric D, Reynolds R, Friede T, Nicholas R (2017). Patient-reported outcomes and survival in multiple sclerosis: a 10-year retrospective cohort study using the multiple sclerosis impact Scale–29. PLoS Med.

[14] Gilmore KJ, Corazza I, Coletta L, Allin S. The uses of patient reported experience measures in health systems: a systematic narrative review. Health Policy. 2022. 10.1016/j.healthpol.2022.07.008 35934546

[15] Cuthbertson L (2015). Patient-centred measurement in British Columbia: statistics without the tears wiped off. Healthc Pap.

[16] Lavallee DC, Chenok KE, Love RM, Petersen C, Holve E, Segal CD (2016). Incorporating patient-reported outcomes into health care to engage patients and enhance care. Health Aff.

[17] Krawczyk M, Sawatzky R, Schick-Makaroff K, Stajduhar K, Öhlen J, Reimer-Kirkham S (2019). Micro-meso-macro practice tensions in using patient-reported outcome and experience measures in hospital palliative care. Qual Health Res.

[18] Howard AF, Medhurst K, Manhas DS, Yang LY, Brown S, Brown E (2021). The usefulness of patient-reported outcomes and the influence on palliative oncology patients and health services: a qualitative study of the prospective outcomes and support initiative. Cancer Nurs.

[19] Aaronson N, Choucair A, Elliott T, Greenhalgh J, Halyard M, Hess R, et al. User’s guide to implementing patient-reported outcomes assessment in clinical practice. International Society for Qual Life Res; 2011. 10.1007/s11136-011-0054-x 22048932

[20] Stover AM, Haverman L, van Oers HA, Greenhalgh J, Potter CM (2021). Using an implementation science approach to implement and evaluate patient-reported outcome measures (PROM) initiatives in routine care settings. Qual Life Res.

[21] Chan EK, Edwards TC, Haywood K, Mikles SP, Newton L (2019). Implementing patient-reported outcome measures in clinical practice: a companion guide to the ISOQOL user’s guide. Qual Life Res.

[22] Cuthbertson L, Dixon D, Schmidt F, Sawatzky R. Towards a working-definition of patient-centred measurement: a review of literature. 2023 10.6084/m9.figshare.22228072.v1

[23] Research AIf. Principles for Making Health Care Measurement Patient-Centered. 2017.

[24] Cuthbertson L, Sawatzky R. Advancing the science of patient-centered measurement methods. https://www.youtube.com/watch?v=X0CSolPPzso (2021). Accessed April 11 2023.

[25] Cuthbertson L, Sawatzky R. Priorities for advancing methods for measuring what matters: patient and researcher perspectives. Qual Life Res. 2021: p. S11–2.

[26] Canadian Institute of Health Research. Strategy for Patient-Oriented Research. http://www.cihr-irsc.gc.ca/e/45851.html (2019). Accessed August 12 2019.

[27] Morse JM, Field PA (1995). Qualitative research methods for health professionals.

[28] Charmaz K. Constructing grounded theory: A practical guide through qualitative analysis (Introducing Qualitative Methods Series). 2006.

[29] Strauss A, Corbin J. Basics of qualitative research: techniques and procedures for developing grounded theory. Sage Publications, Inc; 1998.

[30] Pezold ML, Pusic AL, Cohen WA, Hollenberg JP, Butt Z, Flum DR (2016). Defining a research agenda for patient-reported outcomes in surgery: using a Delphi survey of stakeholders. JAMA Surg.

[31] Coens C, Pe M, Dueck AC, Sloan J, Basch E, Calvert M (2020). International standards for the analysis of quality-of-life and patient-reported outcome endpoints in cancer randomised controlled trials: recommendations of the SISAQOL Consortium. Lancet Oncol.

[32] Mazariego C, Jefford M, Chan RJ, Roberts N, Millar L, Anazodo A (2022). Priority recommendations for the implementation of patient-reported outcomes in clinical cancer care: a Delphi study. J Cancer Surviv.

[33] Brichetto G, Zaratin P (2020). Measuring outcomes that matter most to people with multiple sclerosis: the role of patient-reported outcomes. Curr Opin Neurol.

[34] Pawson R, Tilley N (1997). Realistic evaluation. Vol Book, whole.

[35] Greenhalgh J, Long AF, Flynn R (2005). The use of patient reported outcome measures in routine clinical practice: lack of impact or lack of theory?. Soc Sci Med.

[36] Lordon RJ, Mikles SP, Kneale L, Evans HL, Munson SA, Backonja U (2020). How patient-generated health data and patient-reported outcomes affect patient–clinician relationships: a systematic review. Health Inf J.

[37] Meadows K (2022). Do patient-reported outcome measures tell us the full story?.

[38] Calvert MJ, Cruz Rivera S, Retzer A, Hughes SE, Campbell L, Molony-Oates B et al. Patient reported outcome assessment must be inclusive and equitable. Nat Med. 2022:1–5. 10.1038/s41591-022-01781-8 35513530

[39] Perfetto EM, Oehrlein EM, Love TR, Schoch S, Kennedy A, Bright J. Patient-Centered Core Impact Sets: What They are and Why We Need Them. The Patient-Patient-Centered Outcomes Research. 2022:1–9. 10.1007/s40271-022-00583-x PMC9584872 35653038

[40] Greenhalgh J, Dalkin S, Gibbons E, Wright J, Valderas JM, Meads D (2018). How do aggregated patient-reported outcome measures data stimulate health care improvement? A realist synthesis. J Health Serv Res Policy.

